# Comparative Study of Four Coloured Nanoparticle Labels in Lateral Flow Immunoassay

**DOI:** 10.3390/nano11123277

**Published:** 2021-12-02

**Authors:** Shyatesa C. Razo, Anastasiya I. Elovenkova, Irina V. Safenkova, Natalia V. Drenova, Yuri A. Varitsev, Anatoly V. Zherdev, Boris B. Dzantiev

**Affiliations:** 1 A.N. Bach Institute of Biochemistry, Research Centre of Biotechnology of the Russian Academy of Sciences, Leninsky Prospect 33, 119071 Moscow, Russia; kish218@gmail.com (S.C.R.); elovenkova.anastasiya@yandex.ru (A.I.E.); safenkova@inbi.ras.ru (I.V.S.); zherdev@inbi.ras.ru (A.V.Z.); 2 Agrarian and Technological Institute, RUDN University, Mikluho-Maklaya Street 8/2, 117198 Moscow, Russia; 3 All-Russian Plant Quarantine Centre, Pogranichnaya Street 32, Bykovo-2, Moscow Region, 140150 Moscow, Russia; drenova@mail.ru; 4 Lorch All-Russian Potato Research Institute, Lorch Street 23, Kraskovo, Moscow Region, 140051 Moscow, Russia; varyuriy@yandex.ru

**Keywords:** coloured nanoparticles, lateral flow immunoassay, Au nanoparticles, AuPt nanoparticles, latex beads, magnetic beads, *Erwinia amylovora*

## Abstract

The detection limit of lateral flow immunoassay (LFIA) is largely determined by the properties of the label used. We compared four nanoparticle labels differing in their chemical composition and colour: (1) gold nanoparticles (Au NPs), red; (2) Au-core/Pt-shell nanoparticles (Au@Pt NPs), black; (3) latex nanoparticles (LPs), green; and (4) magnetic nanoparticles (MPs), brown. The comparison was carried out using one target analyte—*Erwinia amylovora*, the causal bacterial agent of fire blight. All nanoparticles were conjugated with antibodies through methods that provide maximum functional coverage like physical adsorption (Au NPs, Au@Pt NPs) and covalent bonding (LPs, MPs). All conjugates demonstrated the same ability to bind with *E. amylovora* through enzyme-linked immunosorbent assay where optical properties of the nanoparticles do not determine the registered signal. However, half-maximal binding was achieved at different numbers of nanoparticles because they differ in size. All conjugates based on four nanoparticle labels were used for lateral flow assays. As a result, Au@Pt NPs provided the minimal detection limit that corresponded to 10^3^ CFU/mL. Au NPs and LPs detected 10^4^ CFU/mL, and MPs detected 10^5^ CFU/mL. The results highlight that simply choosing a coloured label can significantly affect the detection limit of LFIA.

## 1. Introduction

The unique physical and chemical properties of nanoparticles make them efficient for different applications in biomedicine, drug delivery, biosensing, food analysis, agriculture, and many other fields. Thus, nanoparticles are used as labels in lateral flow immunoassay (LFIA), which is the most demanded tool for rapid analysis in conditions of limited resources such as equipment, qualified personnel, and special laboratory facilities [1,2].

In most LFIA applications, a test strip is developed as a tool for visual detection. This strip is a complex multi-membrane composite, wherein a certain membrane performs a specific function. Other components of a test strip take part in the affinity and specific target recognition (antibodies or alternate receptors) and in the generation of a visually detectable signal from the formed complexes (label conjugated to biomolecules). The analyte as part of a liquid sample is applied to the test strip and in the course of its movement with the liquid flow forms a complex in the binding zones with receptor biomolecules adsorbed on the membrane and conjugated with a nanoparticle label. The binding zones are stained with a label and thus become visible [3]. Because all components are pre-applied to the test strip, the end user only needs to dip the strip in the solution to be analysed (or drops the sample onto the strip) and visually evaluates the result after 10–15 min.

For most areas of application of lateral flow test strips (medicine, ecological monitoring, agriculture, and others), the detection of analytes in lower concentrations is highly significant [4]. This situation causes intense developments aimed at finding approaches to reach lower detection limits of LFIA (see recent reviews [1,5,6,7,8,9,10] summarizing these activities). A majority of the proposed approaches involve additional processes to enhance (amplify) the detected signals. However, the amplification is accomplished with the use of additional reagents and typically becomes laborious and increases the time of testing. Another way to achieve low detection limits of LFIA is the change and right choice of a nanoscale label that provides a decrease of detected numbers of complexes. Although the row of proposed alternative labels for LFIA is broad: coloured nanoparticles, fluorescent nanoparticles, SERS-active nanomaterials, magnetic nanoparticles, and carbon nanomaterials for electrochemical detection [11], their studies are mainly limited to demonstrating the principal applicability of the new label. A comparison of the available candidate labels in literature is very limited. In some works, the proposed label is compared to the “gold standard”—gold nanoparticles synthesized by the Turkevich-Frens technique [12,13,14,15,16,17]; or variations of one parameter of the same type labels are considered [18,19]. At the same time, nanoparticle screening for the choice of the best label is not set as a task.

Our study is focused on LFIAs with optical (visual or instrumental) detection as the simplest and widespread approach. The main nanoparticle labels used in such LFIAs are nanoparticles of gold and other noble metals, latex particles, magnetic particles, and carbon particles [2,11,20,21,22]. The nature of nanoparticles largely determines their optical properties and minimal quantities, which can be detected on the membrane. The surface plasmon resonance, inherent in nanoparticles based on noble metals, makes significant impact on their optical properties [23,24].

Therefore, the objective of this study was to compare the four main coloured LFIA labels of different chemical nature and colours: (1) gold nanoparticles (Au NPs), red; (2) Au-core/Pt-shell nanoparticles (Au@Pt NPs), black; (3) latex nanoparticles (LPs), green; and (4) magnetic nanoparticles (MPs), brown. These nanoparticles were chosen primarily because they are the most widely used in LFIA and have the following features: (1) materials are easily available or easy to synthesize, (2) distinct colour, which is perfect for visual detection. In addition, plasmonic properties of Au NPs and Au@Pt NPs increase their extinction and by this way improve sensitivities of LFIAs—see [14,18,25,26,27,28]. Aside from the variety of colours of LPs, it is stable, well-dispersed, and easy to conjugate, making them a great tool as labels [29]. MPs are widely used in LFIA as labels for visual detection, especially for assays with concentrating of analytes using a magnet [30].

To achieve our objective, we performed the following tasks: (1) obtaining conjugates of antibodies with nanolabels, (2) determination of size and aggregation for nanoparticles and their conjugates, (3) characterization of the antigen-binding properties of the conjugates, (4) assembly of lateral flow test strips using membranes with different porosities, (5) obtaining concentration dependences for different labels and membranes, and (6) determination of the detection limit for each variant and their comparison.

The study was carried out using one analyte—the bacterium *Erwinia amylovora*, the pathogen of fire blight of plants from the *Rosaceae* family. *E. amylovora* is a quarantine object in many countries and is considered an important threat to apple and pear orchards [31].

## 2. Materials and Methods

### 2.1. Materials

Carboxylated magnetic nanoparticles (MPs) (440 nm Ø) were purchased from Magsphere (Pasadena, CA, USA), carboxylated latex nanoparticles (LPs) (207 ± 2 nm Ø) were from Smart Diagnostics (Moscow, Russia). Tetrachloroauric(III) acid hydrate, sodium hexachloroplatinate(IV) hexahydrate, ascorbic acid sodium salt, sodium citrate dihydrate, bovine serum albumin (BSA), and N-hydroxysulfosuccinimide sodium salt (NHS) were obtained from Sigma-Aldrich (St Louis, MO, USA). 1-ethyl-3-(3-dimethyl aminopropyl) carbodiimide hydrochloride (EDC) was purchased from Thermo Fisher (Waltham, MA, USA). Goat antibodies against rabbit IgG conjugated with peroxidase (anti-rabbit-HRP) was from Medgamal (Moscow, Russia). Recombinant protein A was purchased from Imtek (Moscow, Russia), and ready-to-use TMB substrate solution from Immunotek (Moscow, Russia). All other reactants of analytical grade (salts, acids, alkalis, etc.) were obtained from Chimmed (Moscow, Russia).

Cells of *Erwinia amylovora* strain CFBP 1430 (*Crataegus* sp., Lille, France, 1972) were used in the study. We obtained cells from the bacterial collection of All-Russian Plant Quarantine Centre (Moscow region, Russia). The bacteria were cultivated at 27 °C on King’s B agar. After 24–48 h the bacteria suspended in 50 mM phosphate-buffered saline, pH 7.4, 100 mM NaCl (PBS). For quantitative cells estimation, 10-fold dilutions were plated in three duplicates. The optical density was measured using NanoDrop 2000 (Thermo Scientific, Waltham, MA, USA) at a wavelength of 600 nm and brought to 0.1 (about 2 × 10^9^ CFU/mL). Bacterial suspensions were stored at −20 °C.

Rabbit polyclonal antibodies (pAbs) specific to *E. amylovora* were described in our previous work [32].

### 2.2. Synthesis of Gold Nanoparticles

**Figure 1:**
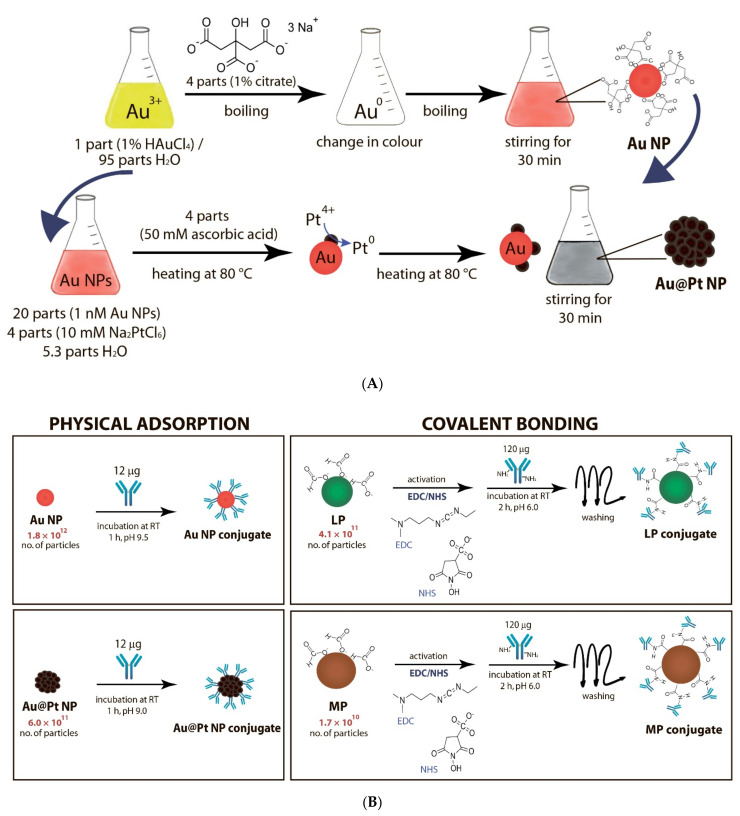
Syntheses of nanoparticles (**A**) and their conjugates with antibodies (**B**). For LPs, a mini centrifuge was used, while for MPs, a magnetic rack was used to remove/separate particles.

Gold nanoparticles (Au NPs) were synthesized (see Figure 1A) using the Frens method [33], with slight changes. One millilitre of 1% HAuCl_4_ was added to 95 mL of deionized water. The mixture was continuously stirred and heated to the boiling point; then, 4 mL of 1% sodium citrate was added. The Au NP solution was continuously boiled for another 30 min, then cooled and stored at 4 °C for future use .

### 2.3. Synthesis of Au@Pt Nanoparticles

Synthesis of Au@Pt NPs was performed following the protocol described by Panferov et al. [25]. Briefly, 20 mL of 1 nM Au NP solution (see Section 2.2) were mixed with 4 mL of 10 mM Na_2_PtCl_6_ solution and 5.3 mL H_2_O for 1 min at 80 ± 2 °C. Then, 4 mL of 50 mM sodium ascorbic salt was added at the rate of 400 μL/min using a peristaltic pump. Then, the mixture was stirred for 30 min at 80 ± 2 °C and further stored at 4 °C (see Figure 1A).

### 2.4. Synthesis of Au-Nanoparticles Conjugates with pAbs

Au NPs were conjugated with pAbs specific to *E. amylovora* following the method described by Razo et al. [34]. The solution of Au NPs was adjusted to pH 9.5. Then, pAbs were added at a ratio equal to 12 µg per 1 mL of Au NPs solution. The synthesis was carried out at RT for 1 h, with continuous mixing using a shaker. BSA as a blocking reagent was added to reach a final concentration of 0.25%. The mixture was centrifuged at 15,000× *g* for 30 min to separate the Au NP conjugates. Afterwards, the synthesized conjugates were resuspended in conjugate buffer (10 mM Tris buffer, pH 7.4, containing 0.25% BSA, 0.05% Tween 20 and 1% sucrose) (see Figure 1B).

### 2.5. Synthesis of Au@Pt Nanoparticles Conjugates with pAbs

Au@Pt NPs were conjugated with pAbs specific to *E. amylvora* through physical adsorption following the method described by Panferov et al. [14,25]. The solution of Au@Pt NPs was adjusted to pH 9.0. 12 µg pAbs was added to each 1 mL of Au@Pt solution (1 nM). The synthesis was carried out at RT for 1 h, with continuous mixing. Afterwards, BSA was added to a final concentration equal to 0.25%. The mixture was centrifuged at 15,000× *g* for 30 min to separate the Au@Pt NP conjugates. The synthesized Au@Pt NP conjugates were resuspended in conjugate buffer (see Figure 1B).

### 2.6. Synthesis of Magnetic Nanoparticle Conjugates with pAbs

The MPs were conjugated with pAbs following the method used by Razo et al. [35] with modifications. In this method, 80 µL of MPs (2.5% *w*/*v*) were mixed in 1520 µL MES buffer (50 mM, pH 6.0), then washed twice with the MES buffer. To activate the carboxyl groups in MPs surface, 500 µL EDC (40 mM) and 500 µL NHS (20 mM) were then added, with continuous mixing at RT for 15 min. Afterwards, MPs were isolated using a MagStand magnetic rack (Evrogen, Moscow, Russia), and washed twice with 900 µL MES buffer (pH 6.0). Then, 400 µL of pAbs (300 µg/mL) were added to the activated MPs. The conjugation process was carried out at RT for 2 h using a shaker. After incubation, unbound particles were removed, and 1 mL ethanolamine (0.5 M) was added to the conjugated MPs to block any active carboxyl groups. The synthesized MP conjugates were suspended in 800 µL conjugate buffer (see Figure 1B).

### 2.7. Synthesis of Latex Nanoparticle Conjugates with pAbs

For the synthesis of LP conjugates with pAbs, 20 µL of LPs (10% *w*/*v*) were mixed with 980 µL of MES buffer (pH 6.0). Washing was done twice with 900 µL MES buffer using a MiniSpin Centrifuge (Eppendorf, Hamburg, Germany). The carboxyl groups of LPs were then activated by the addition of 500 µL EDC (40 mM) and 500 µL NHS (20 mM) for 15 min at RT with shaking, then washed as described above. After washing, 400 µL pAbs (300 µg/mL) was added to the LPs. The mixture was incubated for 2 h at RT in a shaker, and washed, thereafter. To block activated carboxyl groups, 1 mL of ethanolamine (0.5 M) was added as the final step. After final washing, the LP conjugates were resuspended in 800 µL conjugate buffer (see Figure 1B).

For washing, the MiniSpin Centrifuge (Eppendorf, Hamburg, Germany) was used at 12,100× *g* for 15 min per wash. Sonication using a Vibra Cell (Sonics, Newtown, CT, USA) was applied for every stage of the synthesis.

### 2.8. Dynamic Light Scattering (DLS)

The hydrodynamic sizes of the nanoparticles and their conjugates were measured using Zetasizer Nano (Malvern Panalytical, Malvern, UK). All measurements were performed at 25 °C, and scattering angle was equal to 173°. Polydispersity or corresponding % polydispersity (coefficient of variation) was automatically calculated from Cumulants analysis using Zetasizer Software v. 8.00 (Malvern Panalytical, Malvern, UK), and the parameter describes the relative width of the assumed Gaussian distribution.

### 2.9. Transmission Electron Microscopy (TEM)

All nanoparticles were characterized by JEM CX-100 transmission electron microscope (Jeol, Tokyo, Japan) at an accelerating voltage of 80 kV. Nanoparticles were dropped onto a grid (300 mesh, PELCO Grids, Ted Pella, Redding, CA, USA) coated with polyvinyl formal support film. The obtained images of nanoparticles were digitalized and processed with Image Tool software (University of Texas Health Science Center in San Antonio, TX, USA). To estimate the size distribution using the TEM data, single-peaked Gauss approximation was used. The fitting was calculated using OriginPro 9.0 software (OriginLab, Northampton, MA, USA). Meanwhile, using the image of nanoparticles from TEM micrographs, we calculated the ellipticity coefficient by the ratio of major axis to its minor axis. For each type of nanoparticle, mean values and standard deviations of coefficient of ellipticity were found.

### 2.10. Preparation of Test Strips

The test strips prepared for this study had two components—a working nitrocellulose membrane and an adsorbent pad attached to a plastic backing. Absorbent pad (AP045) and plastic backing to attach the membranes (Laminate Type L-P25) were purchased from Advanced Microdevices (Ambala Cantt, Haryana, India). Nitrocellulose membranes (UniSart CN 95, CN 140, CN 180) were purchased from Sartorius (Göttingen, Germany).

The reagents used to form a line for test and control zones on the nitrocellulose membranes were dispensed using an IsoFlow dispenser (Imagene Technology, Lebanon, NH, USA). For the test zone, pAbs specific to *E. amylovora* were adsorbed at a concentration of 1 mg/mL. For the control zone, protein A was adsorbed at a concentration of 0.5 mg/mL. The membranes were dried at 37 °C for 2 h, assembled to multi-membrane composite and cut into strips (3 mm width per strip) using Automatic Cutter ZQ2002 (Shanghai Kinbio Tech, Shanghai, China).

### 2.11. Comparison of Nanoparticle Labels Using Enzyme-Linked Immunosorbent Assays (ELISA)

Bacterial cells (1 × 10^8^ CFU/mL) in PBS were adsorbed in a microplate (Corning Costar Assay Plate) overnight at 4 °C. Afterwards, the microplate was washed with PBST using Thermo Scientific Wellwash (4 cycles, 300 µL PBST/well). After washing, dilutions of nanoparticle labels (diluted from 50 to 10^5^ times) were added to the microplate. Then particles were incubated for 1 h at 37 °C. After incubation, washing was done as described above. Anti-rabbit-HRP was added to each well for detection of the formed complex, incubated for 1 h at 37 °C and washed thereafter. To assess the results, TMB substrate was added to each well and incubated for 15 min at RT. The chemical reaction was stopped by the addition of 1M H_2_SO_4_. The results were analysed by a microplate spectrophotometer, Zenyth 3100 (Anthos Labtec Instruments, Wals, Austria) at A_450_. For further data analysis, OriginPro 9.0 software (OriginLab, Northampton, MA, USA) was used.

### 2.12. Comparison of Nanoparticle Labels Using Lateral Flow Immunoassay (LFIA)

First, to ensure uniformity, we prepared a sufficient volume of bacterial samples (from 1 × 10^2^ to 1 × 10^8^ CFU/mL) to be used for all experiments. For one LFIA test strip, a specific volume of nanoparticle label was mixed to 100 µL of bacterial solution in PBST. MPs were added from 3 to 20 µL, LPs were added from 0.5 to 20 µL, Au NPs were added from 3 to 10 µL, and Au@Pt NPs were added from 1 to 10 µL. Then, test strips were dipped, and results were analysed after 20 min. Afterwards, the results were visible by the naked eye and test strips were scanned using a Canon 9000F Mark II scanner (Canon, Tokyo, Japan). Then, the obtained digital data were processed using TotalLab TL120 (Nonlinear Dynamics, Newcastle, UK) to obtain the values of colour intensities in the test zones of test strips. The quantitative dependences of colour intensity from bacterial cell concentration were plotted using OriginPro 9.0 software (Origin Lab, Northampton, MA, USA). The three-sigma method was used to determine the detection limit.

## 3. Results and Discussion

### 3.1. Synthesis and Characterization of Nanoparticle Labels and Their Conjugates

Two of the four selected labels (Au NPs and Au@Pt NPs) were synthesized (see Figure 1A) in the course of the present work, and two others were commercial ones. For LFIA, a wide range of Au NP’s sizes are used. Most research selected the AuNP with a 20–40 nm diameter for LFIA [36]. As well as Au NPs and Au@Pt NPs synthesized on their basis were simultaneously used, it was important to use exactly those Au NPs that were part of the Au@Pt NPs. According to the selected protocols [14,28], the core of Au@Pt NPs was small Au NPs (not more than ~20 nm).

**Figure 2:**
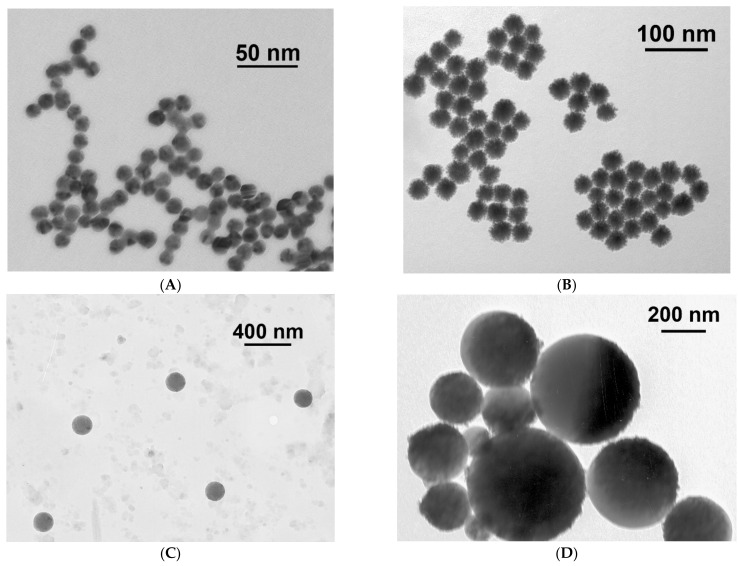
Microphotographs of nanoparticles obtained through TEM. (**A**) Au NPs, (**B**) Au@Pt NPs, (**C**) LPs, (**D**) MPs.

**Figure 3:**
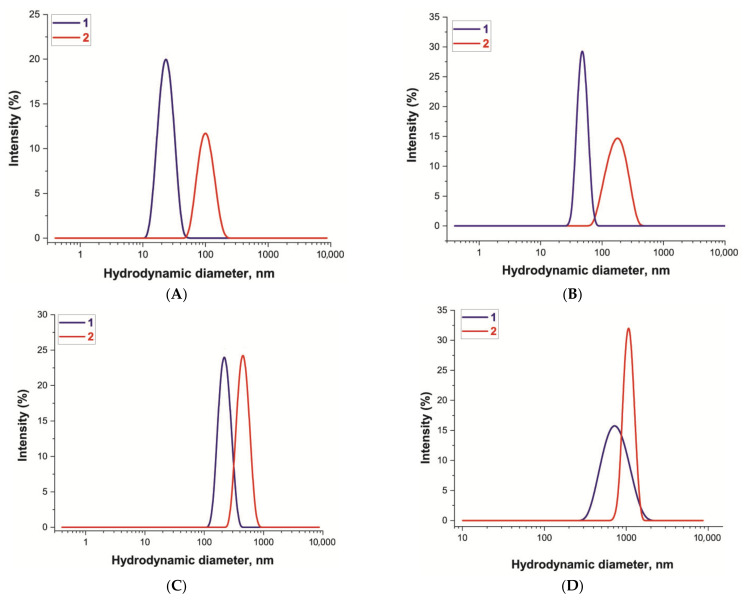
Distribution of hydrodynamic diameters of nanoparticles (1) and their conjugates with pAbs (2) obtained through DLS. (**A**) Au NPs, (**B**) Au@Pt NPs, (**C**) LPs, (**D**) MPs.

The sizes and homogeneity of the synthesized preparations were determined by TEM and DLS. For Au NPs, the TEM obtained an average diameter of 14.4 ± 1.3 nm (Supplementary Materials (SM), Figure S1A), the shape of the particles was determined as spherical, the ellipticity coefficient was equal to 1.1 ± 0.4 (Figure 2A), and the DLS method showed that the hydrodynamic diameter was 22 ± 6.2 nm, polydispersity was 14.7% (Figure 3A). Hereinafter, for other particles, a % polydispersity that was less than 20% polydispersity indicated the monodispersed distributions of nanoparticles [37]. The elliptical coefficients that were close to 1, indicated a spherical shape of nanoparticles.

Au NPs with the resulting characteristics are expected to have a red colour and maximum absorbance at a wavelength of 521 nm. The obtained data corresponded to known specifics. The colour of Au nanoparticle can vary from purple or red depending on the shape, size, and aggregation [24]. According to Iqbal et al. [38], an increase of spherical Au NPs diameter from 8 to 73 nm leads to a shift of the absorption maximum to longer wavelengths from 518 to 545 nm, and the dependence has a linear fit. See also [39] with detailed quantitative evaluation of this trend.

Au@Pt NPs were synthesized using the Au NPs described above, and the reduction of Pt ions on the nanoparticle surface led to the formation of an urchin-type structure. TEM showed an average diameter of 34.3 ± 2.8 nm (Figure S1B), the shape of the particles was determined as spherical and urchin-like, the coefficient of ellipticity was 1.1 ± 0.2 (Figure 2B), and DLS showed that the hydrodynamic diameter was 48.4 ± 8.2 nm, polydispersity was 16.8% (Figure 3B). The Au@Pt NPs preparation had a pronounced black colour and no absorption peaks in the range from 300 to 900 nm. For both types of the synthesized nanoparticles, there were no aggregates according to particle distribution obtained by DLS.

For the preparations of the selected commercial MPs and LPs, the particle diameter and features were also verified by TEM and DLS. As a result, a narrower size distribution was shown for LPs (Figure 2C and Figure 3C) with an average hydrodynamic dimension of 225.7 ± 31.0 nm, polydispersity was equal to 13.7%, and ellipticity coefficient of 1.02 ± 0.08. Particles of green colour were chosen for this work. For MPs (preparation of brown colour), the following parameters were obtained: mean hydrodynamic diameter of 566 ± 155 nm, polydispersity was equal to 19%, and ellipticity coefficient of 1.04 ± 0.10 (Figure 2D and Figure 3D).

For Au NPs and Au@Pt NPs, a narrow TEM distribution (see Supplementary Materials, Figure S1A,B) was obtained and corresponded to a narrow DLS distribution (Figure 3A,B). Therefore, we used average TEM diameters to calculate the particle concentration. For LPs and MPs, the TEM method showed greater heterogeneity (see Supplementary Materials, Figure S1C,D) than the DLS method (Figure 3C,D). Perhaps this was due to the incomplete sorption of large nanoparticles on the TEM grids. Therefore, TEM results for LPs and MPs were used to confirm the integrity of the nanoparticles, and the concentrations were calculated using the average sizes provided by the manufacturer.

For conjugation with antibodies, two of the most typical protocols were used: (1) physical adsorption of biomolecules on the surface of noble metal nanoparticles (Au NPs, Au@Pt NPs), and (2) covalent immobilization of biomolecules on the surface of carboxylated LPs and MPs using N-hydroxysuccinimide cross-linkers. Physical adsorption is one of the simplest methods to obtain bioconjugates. The method is based on hydrophobic, electrostatic interactions, hydrogen bonds, van der Waals forces, and binding between the conducting electrons of noble metal NPs (in our study these were Au NPs and Au@Pt NPs) and amino acid sulphur atoms of the antibody [40]. It implies a direct attachment and does not require modification of either antibodies or noble metal nanoparticles. Conjugation occurs by simply mixing the two components (nanoparticles, antibody) at optimal pH and ion concentration. This method provides non-oriented but strong binding of antibodies to the surface of noble metal nanoparticles. This is sufficient to obtain an effective functional conjugate for LFIA, which has been confirmed by widely used protocols [2,20]. Moreover, Di Nardo et al. showed that the sensitivity of LFIA is dependent on the amount of the antibody bound to Au NPs rather than on the conjugation method [41]. The covalent immobilization (or covalent bonding) involves the formation of chemical bonds between nanoparticles and antibodies. This immobilization is a time consuming and laborious method comprising several stages to prepare conjugates. This method also does not allow to orient unmodified antibodies on the nanoparticle surface. However, covalent bonding often provides more efficient attachment of antibodies to LPs and MPs that do not possess Au/Pt conducting electrons. Non-covalent antibody adsorption can be reversible. Thus, carboxylated LPs and MPs were covalently conjugated with antibodies, providing, as in the case of physical adsorption, the most complete surface coverage with antibodies [42].

To provide the most effective antigen-binding properties of the conjugates, the numbers of antibodies were chosen based on previous studies describing the optimal antibody/nanoparticle ratios for synthesis [14,25]. For all syntheses, the same pAbs specific to *E. amylovora* were used. The changes in the hydrodynamic sizes of the conjugates relative to the particles themselves were observed for all types of nanoparticles (see Figure 3) to confirm the effectiveness of the conjugations performed. For synthesized conjugates of all nanoparticle labels, the average hydrodynamic diameter increased up to the following values: 97 nm for Au NPs, 181 nm for Au@Pt NPs, 470 nm for LPs, and 1155 nm for MNPs. The increase was more than the hydrodynamic size of immobilized IgG molecules. This was consistent with the data that the hydrodynamic diameters of particles are greatly influenced not only with the immobilized molecules, but also due to the environment [43]. DLS recognizes this environment as an additional hydrodynamic shell.

**Figure 4:**
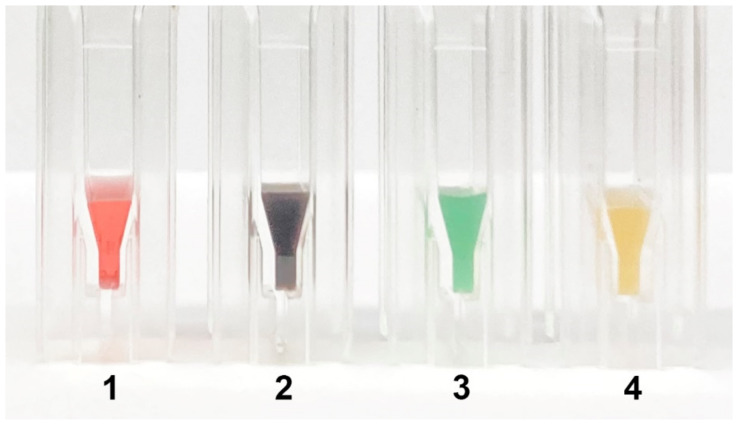
View of synthesized conjugates of pAbs with different nanoparticles: Au NPs (1), Au@Pt NPs (2), LPs (3), MPs (4).

All conjugates had staining corresponding to the colour of the original labels: red (Au NPs), black (Au@Pt NPs), green (LPs), and brown (MPs) (Figure 4).

### 3.2. Comparison of Nanoparticle Labels Using ELISA

The functional characterization of the synthesized conjugates was carried out by a method that does not depend on the optical properties of nanoparticle labels; hence, the ELISA method was chosen. ELISA assumes sequential binding of reagents under equilibrium conditions and reflects the assembly of complexes under the same conditions. That means the antigen (*E. amylovora* cells, 10^8^ CFU/mL) was adsorbed on the microplate surface, the pAbs-nanoparticle conjugate was introduced and after the formation of immune complexes with bacteria and washing of unbound conjugates, complexes were detected with conjugate of anti-species (antirabbit) antibodies with peroxidase. The main factor influencing the generated signal was the antigen-binding capacity of the conjugate. In ELISA the optical characteristics and the nature of each nanoparticle label does not affect the signals. The resulting quantitative dependences reflect the efficiency of conjugate binding. ELISA makes it possible to evaluate the efficiency of conjugates obtained by the physical adsorption and by the covalent immobilization.

**Figure 5:**
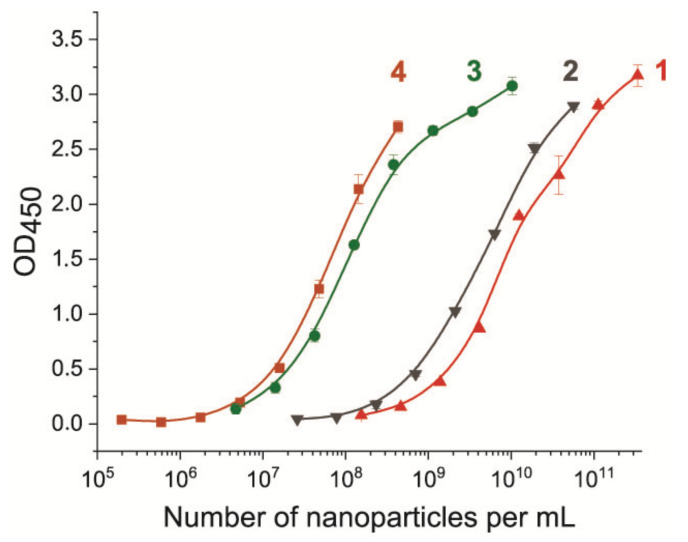
Binding dependencies of the pAb-nanoparticle conjugates to the bacterial cell (10^8^ CFU/mL of *E. amylovora*) adsorbed on the microplate surface. Numbers correspond to the type of nanoparticles: 1—Au NPs, 2—Au@Pt NPs, 3—LPs, 4—MPs.

Initially, all conjugates were added in a dilution range from 50-fold to 10^5^-fold. For correct interpretation, the results were recalculated into other units—the number of nanoparticles per mL. The resulting dependencies are shown in Figure 5. All conjugates showed binding to the bacteria. The binding dependencies had similar graphs for all four nanoparticle labels, and the slopes of the curves were the same, which was a consequence of the presence of the same pAbs in the conjugates. However, the half-maximal binding was achieved at different numbers of nanoparticles because all nanoparticle labels differed in size. In the series with increasing sizes of Au NPs, Au@Pt NPs, LPs, and MPs (Ø 14, 34, 207, 440 nm), smaller particles required a larger number of particles to attain a signal of half-maximal binding compared to larger particles. This effect is the result of the number of antibodies on the nanoparticle surface available for binding to anti-species antibodies. The larger particle carries more antibodies available for binding to anti-species antibodies and, therefore, fewer particles generate a detectable signal. To summarize, the surface area and the number of antibodies attached is directly proportional, regardless of the method of immobilization (physical adsorption or covalent bonding). Thus, both types of immobilizations ensured complete coverage of the surface with antibodies. This result showed that the half-maximal binding varied up to two orders of magnitude in a method that does not depend on the optical properties of nanoparticles.

### 3.3. Comparison of Nanoparticle Labels Using LFIA

LFIA is performed in a flow-through mode and immune complexes are assembled in non-equilibrium conditions—in contrast to ELISA. The LFIA performance depends on many factors such as antigen-binding properties of the antibody and antibody-NP conjugate, type of nanomaterial, size, optical characteristics, porosity of membranes, and flow rate of nanoparticle label in each membrane that could affect the formation of the complex on the test zones. The quantitative dependences obtained in LFIA and ELISA with a dominant antigen-binding factor may differ.

The considered LFIA of *E. amylovora* was realized in a sandwich format with the obtained triple labelled complex at the test zone, namely the immobilized antibodies, analyte, and labelled antibodies. This is the common choice for large multivalent antigens providing better sensitivity. Besides, the sandwich format of LFIA provides a direct dependence of the signal on the analyte concentration and the efficiency of label detection accords directly to the efficiency of detection of the formed immune complexes. The detection limit of LFIA also depends on a combination of factors, in this regard, we compared different parameters of LFIA based on the performance of each nanoparticle label.

For particles of different sizes, working membranes of different porosities are optimal. Three types of membranes with different porosity were used, with manufacturer-provided rates of fluid flow: CN-95 (65–115 s/40 mm capillary speed down web, purified water), CN-140 (90–150 s/40 mm), CN-180 (135–175 s/40 mm). We considered a simple assembly of the test strip containing only nitrocellulose membrane with test and control zones followed by an absorbent membrane. Addition of conjugates to the analysed sample in the course of the assay excluded the necessity of the glass fibre membrane for the conjugate and special tasks associated risks of the uncomplete release of the conjugate. The possibility of excluding the sample membrane arose because the comparison presented in the work is not related to the features of different matrices and the need to separate their components.

**Figure 6:**
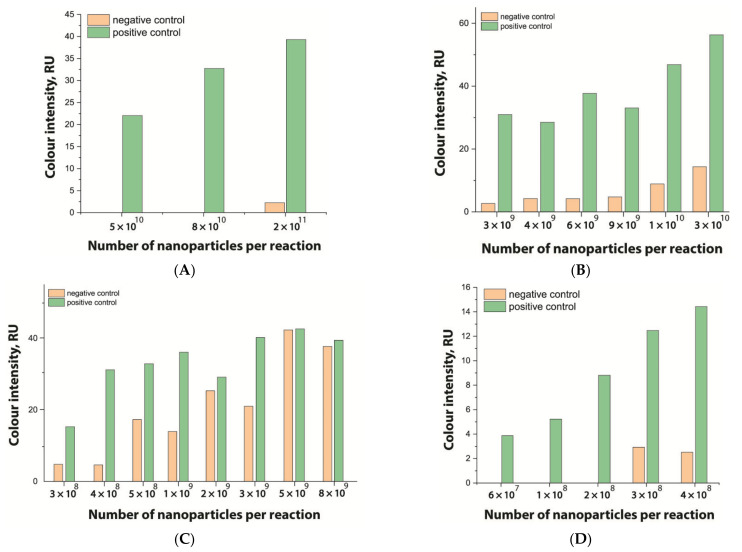
Optimization of the number of nanoparticles per reaction in LFIA based on CN-95 nitrocellulose membrane with (**A**) Au NPs, (**B**) Au@Pt NPs, (**C**) LPs, (**D**) MPs. The negative control is buffer without bacterial cells, and the positive control is buffer with 1 × 10^7^ CFU/mL *E. amylovora*.

At the first stage of comparison, the number of nanoparticles per reaction for each of their conjugates was chosen. For this, a comparison of the signal-to-noise ratios for each conjugate on each type of nitrocellulose membrane was conducted. A typical comparison of the four conjugate variants is shown in Figure 6 as signals obtained from samples without cells and with 1 × 10^7^ CFU/mL *E. amylovora*. For further experiments, the numbers of nanoparticles that provide the maximum signal and maximum signal-to-noise ratio were selected. Thus, for Au NPs these requirements were met by 8 × 10^10^ nanoparticles per reaction (5 μL conjugate) for all types of membrane, for Au@Pt NPs it was 3 × 10^9^ (1.5 μL conjugate), for LPs it was 4 × 10^8^ (0.7 μL), and for MPs it was 2 × 10^8^ (10 μL) only for CN-95 and CN-140 because the porosity of CN-180 was not optimal for MPs. Thus, the larger the particle, the fewer particles that were necessary to maximize the signal-to-noise ratio.

Selected amounts of conjugates were used to detect bacterial cells over a range of concentrations. Table 1 shows the scans of the test strips and the obtained concentration dependences for *E. amylovora*. The brightness of the control and test zones in experiments with different types of nitrocellulose membranes demonstrates that Au NPs and Au@Pt NPs can be used with all types of membranes; for LPs and MPs, the CN-180 membrane did not provide efficient movement of the particles. For Au NPs, the minimum limit of detection (LOD) was 10^4^ CFU/mL *E. amylovora*, for Au@Pt NPs it was 10^3^ CFU/mL, for LPs it was 10^4^ CFU/mL, for MPs it was 10^5^ CFU/mL. Thus, LPs were more efficient than MPs, because the number of particles was only twice the number of particles of MPs providing LOD, which was an order of magnitude lower. The second conclusion from the results was that for Au NPs 8 × 10^10^ particles per reaction provided the same LOD as for LPs, at 4 × 10^8^ particles per reaction. The minimal LOD was shown with Au@Pt using 3 × 10^9^ particles in the reaction, which was 26 times lower than that for AuNPs and 7.5 times higher than that for LPs. The advantages of a black label correlate with previously reported results compared to carbon nanoparticles and Au NPs (~2 times LOD difference), Au@Pt NPs, and Au NPs (2–10 times LOD difference) [14,15].

For all nanoparticle labels, the limitation was the non-specific binding to the membrane, which was observed at high numbers of particles in the reaction. Therefore, although LPs provide better recognition performance than Au@Pt NPs (fewer particles provide the same detection limit), due to background staining, a lower detection limit cannot be achieved.

As a result of the comparison of nanoparticle labels in the LFIA, the prospects of their use as labels with optical properties increase in the series of MPs, Au NPs, LPs, and Au@Pt NPs. In this regard, the method of antibody—NP conjugation (physical adsorption or covalent immobilization) of antibodies on the surface of nanoparticles—did not determine the detection limit of LFIA. As can be seen from the ELISA data (see Section 3.2), the minimum number of particles sufficient for the formation of complexes is required for larger particles, but in LFIA this is not a decisive factor. When comparing four typical nanoparticle labels, it is not size that determines the best sensitivity. In this case, the size influences the choice of membrane porosity. The colour rather than the type of conjugation was found to be the more significant factor. The colour determined to a greater extent the sensitivity of the LFIA. Thus, careful selection of a label for analysis can provide a significant gain in sensitivity.

## 4. Conclusions

The nanoparticles used in LFIA as labels with an optical detection can be of different nature and different colours. It is generally believed that the difference between coloured labels is not very significant. However, this conclusion was based on comparisons in pairs of short rows of similar compounds [14,15,19]. In this work, we compared four types of labels that differ in chemical nature and colour—Au NPs (red), Au@Pt NPs (black), LPs (green), and MPs (brown). Comparison in the LFIA sandwich format showed that the labels differed in their amounts causing the maximal signal-to-noise ratio. At high numbers of particles in the reaction, nonspecific binding to the membrane was observed for all considered nanoparticles. Depending on the type of nanoparticles, the number of particles per synthesis was from 2 × 10^8^ (MPs) to 8 × 10^10^ (Au NPs). At the same time, the minimum detection limit (10^3^ CFU/mL *E. amylovora*) was obtained for Au@Pt NPs, which in the reaction was 26 times lower than for AuNPs, 7.5 times higher than for LPs, and 15 times higher than for MPs.

Based on the results obtained we can confirm the assumption that the black label is likely the most effective colour marker for detection on test strips. This is consistent with van Amerongen’s comments on the advantages in LFIA of other black nanoparticles, carbon ones [44]. The promise of LFIA labels with optical properties increases in the series of MPs, Au NPs, LPs, and Au@Pt NPs. The difference in the detection limits in this series reached two orders of magnitude, which is comparable with typical enhancements reached by amplification [5,8,45].

## Figures and Tables

**Table 1:** Comparison of test strips based on different nitrocellulose membranes and different nanoparticle labels.

	Test Strip Appearance		Dependencies of Colour Intensity in the Test Zone of LFIA from a Dilution of the Sample Spiked with *E. amylovora*
Au NPs	CN-95	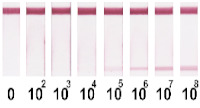	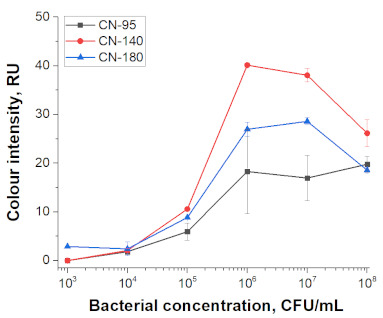
	CN-140	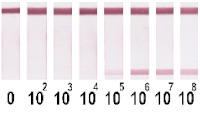	
	CN-180	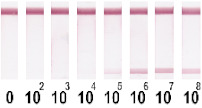	
Au@Pt NPs	CN-95	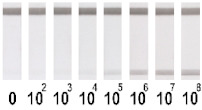	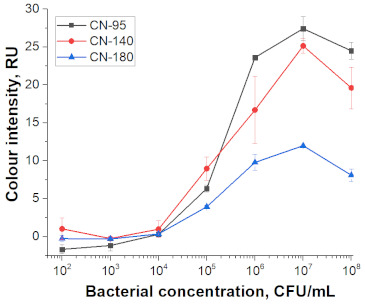
	CN-140	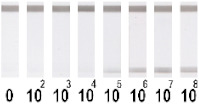	
	CN-180	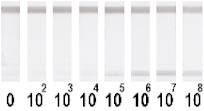	
LPs	CN-95	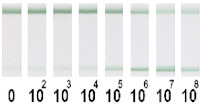	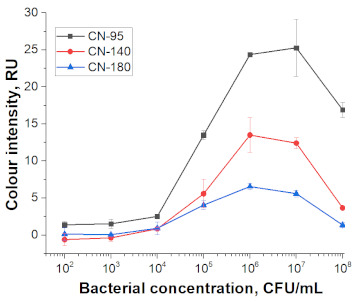
	CN-140	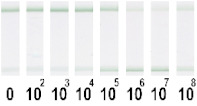	
	CN-180	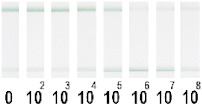	
MNPs	CN-95	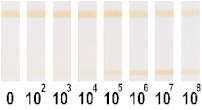	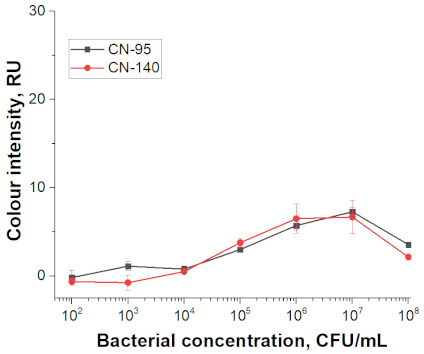
	CN-140	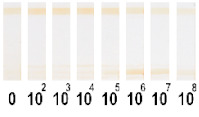	
	CN-180	N/A	

## Data Availability

The data presented in this study are available on request from the corresponding author.

## Supplementary Materials

The following are available online at https://www.mdpi.com/article/10.3390/nano11123277/s1, Figure S1: Size distributions of nanoparticles by TEM. (A) Au NPs, total number of counts (n) = 119; (B) Au@Pt NPs, n = 110; (C) LPs, n = 100; (D) MPs, n = 55.
