# Supplementary material for: Comparative Study of Four Coloured Nanoparticle Labels in Lateral Flow Immunoassay
Source: Nanomaterials (Basel). 2021 Dec 2;11(12):3277. doi: 10.3390/nano11123277 (PMC8708713; doi:10.3390/nano11123277)
Supplement: Supplementary file 1 [file nanomaterials-11-03277-s001.zip › nanomaterials-1472287-supplementary.pdf]

Supplementary Materials

Comparative study of four coloured nanoparticle labels in lateral flow immunoassay

Shyatesa C. Razo [1, 2], Anastasiya I. Elovenkova [1], Irina V. Safenkova [1], Natalia V. Drenova [3], Yuri A. Varitsev [4], Anatoly V. Zherdev [1] and Boris B. Dzantiev [1,*]

[1]A.N. Bach Institute of Biochemistry, Research Centre of Biotechnology of the Russian Academy of Sciences, Leninsky Prospect 33, 119071 Moscow, Russia
[2]Agrarian and Technological Institute, RUDN University, Mikluho-Maklaya Street 8/2, 117198 Moscow, Russia
[3]All-Russian Plant Quarantine Centre, Pogranichnaya Street 32, Bykovo, Ramenskoe, Moscow Region, 140150, Russia
[4]Russian Potato Research Center, Kraskovo, Moscow Region, 140051, Russia
*****Correspondence: dzantiev@inbi.ras.ru; Tel.: +74959543142

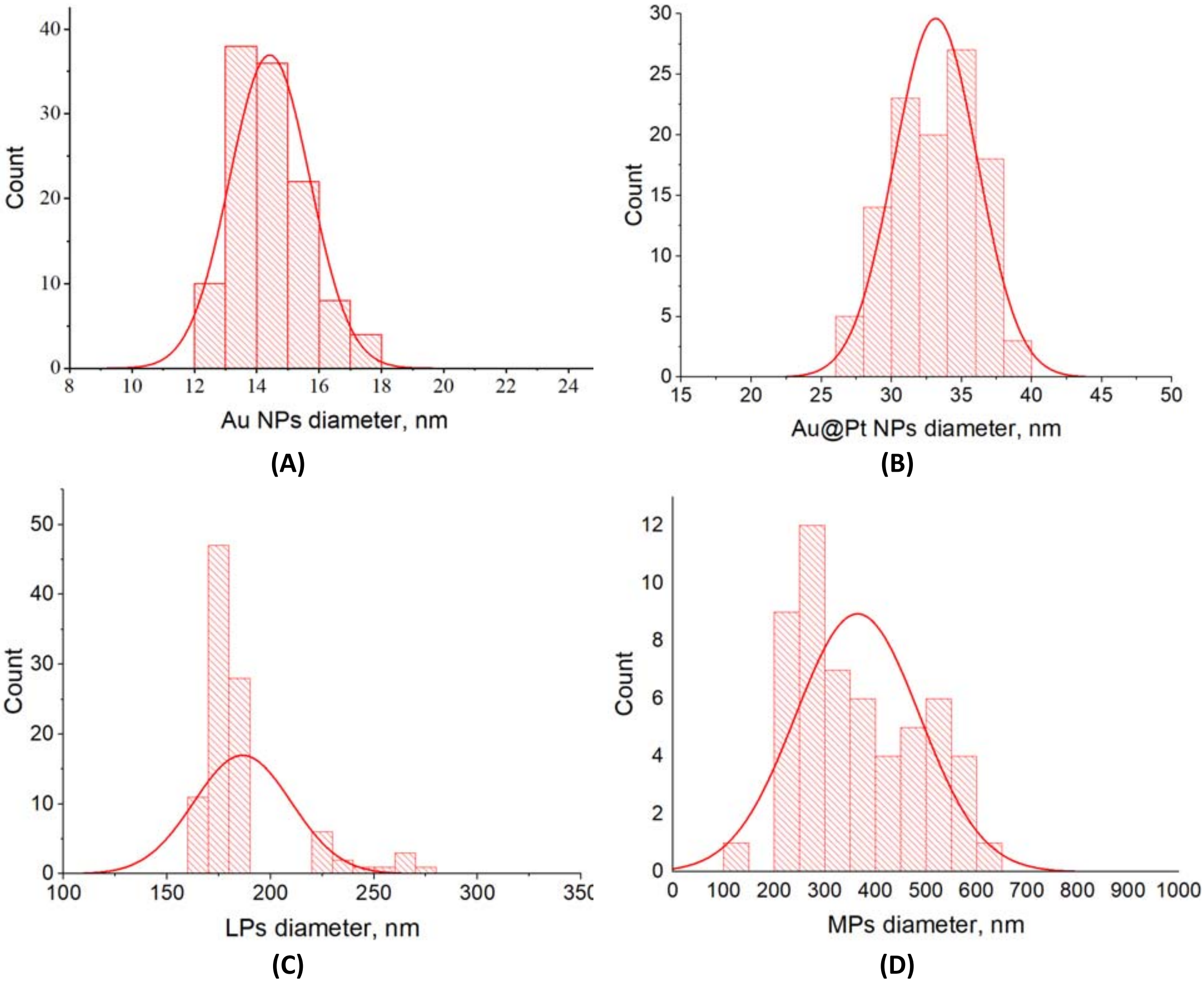

Figure S1. Size distributions of nanoparticles by TEM. (A) Au NPs, total number of counts (n) = 119; (B) Au@Pt NPs, n = 110; (C) LPs, n = 100; (D) MPs, n = 55.
